# Pattern of Adaptive Divergence in *Zingiber kawagoii* Hayata (Zingiberaceae) along a Narrow Latitudinal Range

**DOI:** 10.3390/plants11192490

**Published:** 2022-09-23

**Authors:** Yi-Shao Li, Pei-Chun Liao, Chung-Te Chang, Shih-Ying Hwang

**Affiliations:** 1 School of Life Science, National Taiwan Normal University, 88 Tingchow Road, Section 4, Taipei 11677, Taiwan; 2 Department of Life Science, Tunghai University, 1727 Taiwan Boulevard, Section 4, Taichung 40704, Taiwan

**Keywords:** adaptive divergence, AFLP, allele frequency, annual temperature range, latitudinal gradient, population mean *F*_ST_, *Zingiber kawagoii*

## Abstract

Ecological and evolutionary processes linking adaptation to environment are related to species’ range shifts. In this study, we employed amplified-fragment-length-polymorphism-based genome scan methods to identify candidate loci among *Zingiber kawagoii* populations inhabiting varying environments distributed at low to middle elevations (143–1488 m) in a narrow latitudinal range (between 21.90 and 25.30° N). Here, we show evidence of selection driving the divergence of *Z. kawagoii*. Twenty-six *F*_ST_ outliers were detected, which were significantly correlated with various environmental variables. The allele frequencies of nine *F*_ST_ outliers were either positively or negatively correlated with the population mean *F*_ST_. Using several independent approaches, we found environmental variables act in a combinatorial fashion, best explaining outlier genetic variation. Nonetheless, we found that adaptive divergence was affected mostly by annual temperature range, and it is significantly positively correlated with latitude and significantly negatively correlated with the population mean *F*_ST_. This study addresses a latitudinal pattern of changes in annual temperature range (which ranged from 13.8 °C in the Lanyu population to 18.5 °C in the Wulai population) and emphasizes the pattern of latitudinal population divergence closely linked to the allele frequencies of adaptive loci, acting in a narrow latitudinal range. Our results also indicate environmentally dependent local adaptation for both leading- and trailing-edge populations.

## 1. Introduction

The altitude-for-latitude model projected the scales of range retractions in altitude and latitude due to warming [1]. Locally adaptive alleles associated with environmental conditions in range shift margin populations are essential for species’ future resilience to climate change. Temperature and precipitation are the two most important climatic factors influencing the distribution, differentiation, and diversity of species [2]. Genetically based ecotypes may evolve corresponding to environmental changes and play a role in minimizing the extinction risk [3]. Genetic variation may vary along latitudinal and altitudinal clines [4]. Latitudinal environmental patterns are found to be correlated with intraspecific and interspecific diversity distributed in a large geographic scale [5]. Taiwan only covers a narrow latitudinal range of 385 km, lying between the north latitude 21.90 and 25.30, and the identification of latitudinal patterns in plant diversity and differentiation is important given the presence of ecological heterogeneities due to rugged topography and steep elevation in Taiwan.

**Figure 1:**
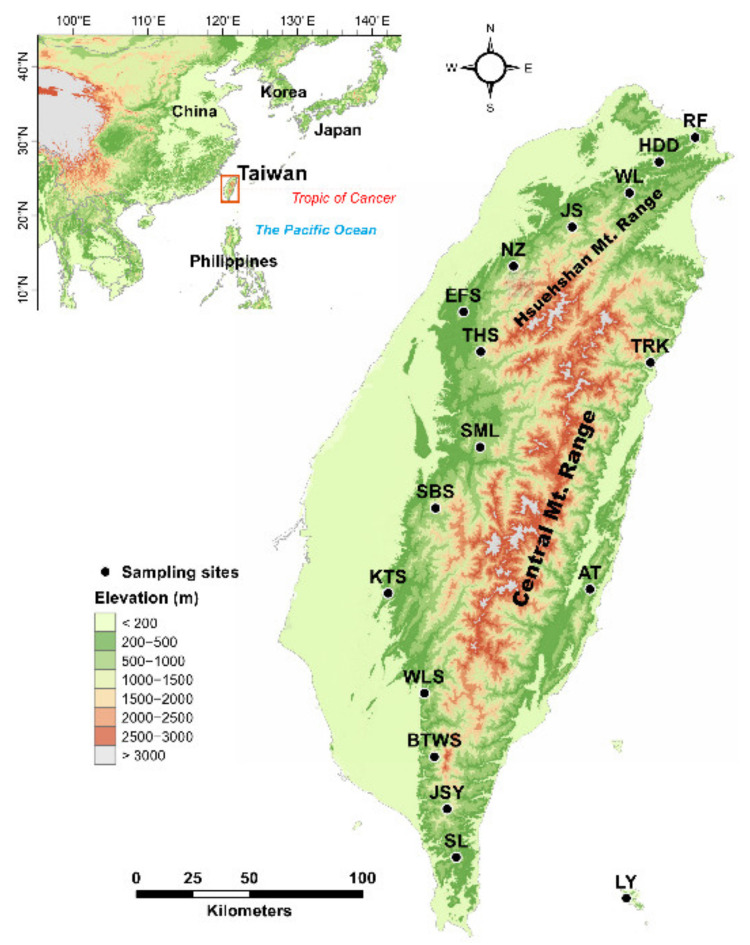
Sampling localities of the 17 *Zingiber kawagoii* populations. The coordinates of sampling sites were used to plot population locations using Tools in ArcGIS v.10.8.1. Map was derived from the default map database in ArcGIS, and the 20 m digital elevation model was used in the generation of elevation gradients. See Table 1 for abbreviations of the population names.

*Zingiber kawagoii* is a species of herbaceous perennial plant in the Zingiberaceae family found in Taiwan and a small offshore island southeast of Taiwan. This species is endemic to Taiwan and is widely distributed along the west sides of the Hsuehshan Mountain Range and the Central Mountain Range in Taiwan, but it is sparsely distributed east of these mountain ranges, at low to middle elevations (140–1500 m), and from the southern to northern tip of Taiwan (Figure 1). *Zingiber kawagoii* was also found on a small island, Lanyu (orchid island), 90 km off the southeast coast of Taiwan. Populations of *Z. kawagoii* are found in different habitats including forest understories, forest edges, slopes, and valleys. Extremely low intraspecific variation and high population differentiation were found based on chloroplast DNA (cpDNA) variation [6]. A mean inbreeding coefficient of 0.656 was found, suggestive of a deficiency of heterozygotes that may have resulted from bottlenecks and/or inbreeding [7]. The latitudinal northerly expansion of *Z. kawagoii* after the last glacial maximum (LGM) has been inferred using ecological niche modeling [8]. This climate-induced northerly expansion may reduce genetic diversity and increase genetic as well as migration load [9,10], limiting the ability for adaptation and persistence in novel environments. However, locally adapted alleles may have been evoked during expansion, encountering novel selective regimes [4,11]. Although drift and migration both decrease local adaptation, smaller range-front populations may develop local adaptive divergence when selection is strong [12]. The leading-edge populations of northerly expanded *Z. kawagoii* may evolve locally adapted alleles in association with a leading-front environment. Nonetheless, trailing-edge populations are also important to the future survival of species [4,11,13].

Lower-latitude populations within species may show greater genetic divergence and evolutionary independence, contributing to reproductive isolation and speciation [5]. Moreover, adaptive genetic variation is widespread in herbaceous species with low levels of gene flow under strong selection pressures [14]. Because of its widespread distribution from the south to the north of Taiwan and the biogeographic history of latitudinal northerly expansion [8], the examination of the latitudinal pattern of *Z.* *kawagoii* population divergence related to environmental gradients will advance our understanding of species’ response to global change, particularly in a geographic area only spanning a narrow latitudinal range. Spatial heterogeneity in populations of *Z. kawagoii* may have played roles in driving local adaptation associated with environmental factors. Although genetic diversity in the latitudinal leading- and trailing-margin populations may be reduced, adaptive loci driven by natural selection may accumulate their frequencies at both range margins of distribution correlated strongly with environment [12,15,16]. No study has been conducted to test for local adaptation in *Z.* *kawagoii*. In this study, we surveyed the genetic variation in 212 individuals from 17 populations of *Z. kawagoii* using amplified fragment length polymorphisms (AFLPs) [17] and collected information regarding the environmental variables of sampling sites. The general aim of this study was to investigate the pattern of population divergence in *Z. kawagoii* related to adaptive evolution along a narrow latitudinal range. Specifically, we asked (1) how do population divergence and environmental variables vary along a narrow latitudinal range? (2) Which environmental variable(s) may have played the main role, and do environmental variables act in a combinatorial fashion in driving adaptive genetic divergence? (3) How do the allele frequencies of adaptive loci vary corresponding to the levels of population divergence?

## 2. Materials and Methods

### 2.1. Sampling, DNA Extraction, and AFLP Genotyping

*Zingiber kawagoii* individuals were collected from 17 populations (*n* = 212) which spanned a latitudinal range of 21.90–5.30° N and an altitudinal range of 143–1488 m (Figure 1). Within each population, samples were collected with a space at least 10 m apart. Because of latitudinal northerly expansion [7], populations distributed in higher latitudes can be recognized as leading-edge populations, and those distributed in the lower latitudes can be recognized as trailing-edge populations. Total genomic DNAs were extracted based on a cetyltrimethyl ammonium bromide (CTAB) procedure [18]. Total genomic DNA was ethanol precipitated and dissolved in 200 µL of TE buffer (pH 8.0). We quantified total genomic DNA using a NanoDrop spectrophotometer (NanoDrop Technology, Wilmington, DE, USA). In a total 10 µL reaction volume, 200 ng of total genomic DNA was mixed with 1 U *Eco*RI and 1 U *Mse*I restriction enzymes incubated in 10X CutSmart buffer (New England Biolabs, Ipswich, MA, USA) at 37 °C for 1.5 h for restriction digestion. The reaction was deactivated at 65 °C for 15 min. The digested DNA products were ligated to AFLP adaptors (5 µM *Eco*RI and 50 µM *Mse*I) with 5 U T4 DNA ligase (Thermo Scientific, Vilnius, Lithuania) and 5X ligation buffer (Thermo Scientific) at 22 °C for 1 h in a 10 µL ligation reaction mixture.

AFLP pre-selective amplification was performed using polymerase chain reaction (PCR). Adaptor ligated product (1:9 dilution with ddH_2_O) was mixed with 12 µM *Eco*RI (E00: 5′-GACTGCGTACCAATTC-3′) primer, 12 µM *Mse*I (M00: 5′-GATGAGTCCTGAGTAA-3′) primer, 2.5 mM dNTPs, 1 U *Taq* DNA polymerase (Zymeset Biotech, Taipei, Taiwan), and 10X PCR buffer (Zymeset) in a 20 µL total volume. The PCR for pre-selective amplification was an initial holding at 72 °C for 2 min and pre-denaturation at 94 °C for 3 min, followed by 25 cycles of 30 s at 94 °C, 30 s at 56 °C, and 1 min at 72 °C, with a final 5 min holding at 72 °C. Either three or five additional bases were added at the ends of E00 and M00, and 90 primer combinations were screened initially for proper amplification, the number of fragments were amplified, and the genotyping error rate did not exceed 5%. Finally, 11 *Eco*RI-*Mse*I (E00 and M00) primer combinations were used in selective amplification (Table S1). A labeled *Eco*RI selective primer (6-carboxyfluorescein or hexachloro-fluorescein dye labeled) was used in selective amplification. Selective amplification was performed in a 20 µL total volume containing 10 µM *Eco*RI and 10 µM *Mse*I primers, 2.5 mM dNTPs, 2 U *Taq* DNA polymerase (Zymeset), 10X PCR buffer (Zymeset), and diluted pre-selective amplified products (1:19 dilution with ddH_2_O). Selective PCR was an initial holding at 94 °C for 3 min, followed by 13 cycles of 30 s at 94 °C, 30 s at 65–56 °C (decreasing the temperature by 0.7 °C each cycle), 1 min at 72 °C, then 23 cycles of 30 s at 94 °C, 30 s at 56 °C, and 1 min at 72 °C, with a final 5 min holding at 72 °C. Selective amplification products were electrophoresed on an ABI 3730XL DNA analyzer. We used Peak Scanner v.1.0 (Applied Biosystem, Foster City, CA, USA) to score amplification fragments. A fluorescent threshold set at 150 units was used to avoid background noise when scoring AFLP fragments in the range of 100–500 bp. We removed low peaks and fragments within one nucleotide in a ±0.8 base pair window which were recognized as the same fragment. Additionally, amplified fragments that scored higher than 99% or less than 1% of individuals were also removed. Three randomly chosen samples in each population were used to calculate the genotyping error rate per locus. Loci with an error rate per locus greater than 5% were removed [19]. The mean error rate per locus was 4.11% (Table S1).

### 2.2. Environmental Variables

Three categories of environmental variables were used in the study. Nineteen bioclimatic variables for sample sites at 30 s spatial resolution (~1 km) were downloaded from the WorldClim 1.4 for information related to temperature and precipitation [20]. Topographic variables, including aspect, elevation, and slope at 20 m resolution were obtained from Taiwan Geospatial One Stop, Ministry of the Interior (https://data.gov.tw/dataset/138563, accessed on 12 June 2021). Apart from the bioclimatic variables downloaded from WorldClim and three topographic variables, twelve other environmental variables not directly defined as bioclimatic and topographic variables were grouped as ecological variables. The twelve ecological variables were the normalized difference vegetation index (NDVI), the enhanced vegetation index (EVI), the leaf area index (LAI), the fraction of absorbed photosynthetically active radiation (fPAR), the relative humidity (RH), cloud cover (CLO), sunshine hours (SunH), the number of rainfall days per year (RainD), the mean wind speed (WS_mean_), the soil pH, the annual total potential evapotranspiration (PET), and the annual moisture index (MI).

Data from a moderate resolution imaging spectroradiometer (MODIS) recorded during 2001–2020 in the Land Process Distributed Active Archive Center (http://lpdaac.usgs.gov, accessed on 12 June 2021) were obtained for NDVI and EVI (dataset MOD13A2, 1 km resolution), LAI and fPAR (MOD15A2 dataset, 500 m resolution), and PET (MOD16A3 dataset, 500 m resolution). The monthly mean values of these variables were computed using a maximum-value composite procedure. The monthly mean values of RH, CLO, SunH, RainD, and WS_mean_ at 1 km resolution were calculated using a universal spherical model of the Kriging method in ArcGIS with data obtained from the Data Bank for Atmospheric & Hydrologic Research (https://dbahr.pccu.edu.tw/, recorded in 1991–2020; accessed on 12 June 2021). Sampling site soil pH values were obtained from the soil data of an island-wide, 1,150 site investigation conducted in 1969–1986 by the Agriculture and Food Agency of Taiwan. Annual potential evapotranspiration values derived from the annual mean temperature and annual precipitation were used to calculate the annual MI.

Environmental variables of the three categories were separately used in the calculation of variance inflation factor (VIF) with a correlation threshold of |0.7| using the *vifcor* function of the R package usdm [21] in the R environment [22]. The bioclimatic variables BIO7 (annual temperature range), BIO9 (mean temperature of the driest quarter), BIO12 (annual precipitation), and BIO19 (precipitation of the coldest quarter); topographic variables aspect, elevation, and slope; and the ecological variables CLO, EVI, LAI, NDVI, MI, PET, RH, soil pH, and WS_mean_ were retained based on VIF values smaller than 10. Moreover, the forward selection procedure was used for these variables in each environmental category separately, and the final set of environmental variables retained were aspect, BIO7, BIO12, NDVI, PET, RH, and WS_mean_ (Table 1) if more than 5% of outlier genetic variation (adjusted *R*^2^ ≥ 0.05) was explained by the individual variable (see forward selection below, Table S2). Pearson’s correlation coefficients of pairwise comparisons and VIFs (all < 5) are reported in Table S3.

### 2.3. Genetic Diversity

AFLP-SURV v.1.0 [23] software was used to estimate the population unbiased expected heterozygosity (*uH*_E_) [24] and the proportion of polymorphic loci (%*P*, 95% criterion) based on allele frequencies using the settings of the Hardy–Weinberg equilibrium and non-uniform prior distribution. The per locus *uH*_E_ was estimated using ARLEQUIN v.6.0 [25]. The index of association *I*_A_ [26] and the modified index of association (*r*_D_) [27] are measures of multilocus linkage disequilibrium. These two measures were calculated using the *ia* function of the R package poppr [28]. A linear mixed effect model (LMM) was used to estimate the difference of the mean *uH*_E_ per locus among and between populations. In LMMs, population and locus were used as a fixed factor and a random factor, respectively, and they were analyzed using the *lmer* function of the R package lme4 [29] based on the reduced maximum likelihood method. Significance tested using the *Anova* function of the R package car was based on type II Wald *χ*^2^ statistics [30]. Pairwise population comparisons of the mean *uH*_E_ per locus with Tukey’s post hoc test were assessed using the *lsmeans* function of the R package emmeans [31].

### 2.4. Genetic Differentiation, Clustering, and Relationships

The analysis of molecular variance (AMOVA) was used to estimate the level of genetic differentiation between populations (*Φ*_ST_) using the *poppr.amova* function of the R package poppr. Significance was tested using the *randtest* function of the R package ade4 [32] with 9999 permutations. The pairwise population *F*_ST_ was computed using ARLEQUIN, and the significance was tested with 10,000 permutations. Additionally, the level of divergence for each population from the remaining populations was calculated as the mean value of the pairwise *F*_ST_ for each population against the rest of the populations (denoted as the population mean *F*_ST_). The population mean *F*_ST_ can be used as a proxy of the level of one population diverging genetically from the remaining populations.

Genetic homogeneous groups of individuals were assessed using the sNMF algorithm of landscape and ecological association (LEA) [33] and discriminant analysis of principal components (DAPC) [34]. A clustering scenario of *K* = 1–18 based on least-squares optimization was estimated using the *snmf* function of the R package LEA [33]. The parameters including regularization, iterations, and repetitions in *snmf* were set to 100, 200, and 10, respectively, and other arguments were set to defaults. The *find.clusters* and *dapc* functions of the R package adegenet [35] were used in DAPC analysis setting *K* = 1–10. The mean minimal cross-entropy (CE) in LEA and the Bayesian information criterion (BIC) in DAPC were estimated to determine the optimal number of clusters.

A neighbor-joining (NJ) tree was used to assess the genetic relationships among individuals. The *nei.dist* function of the R package poppr was used to calculate pairwise Nei’s genetic distances [36]. Nei’s genetic distance matrix was used to generate an unrooted NJ tree using the *nj* function of the R package ape [37], and bootstrap support values (BSP) were calculated based on 1000 replicates using the *aboot* function of the R package poppr.

### 2.5. Test for F_ST_ Outliers

To detect the signature of selection on AFLP loci, *F*_ST_ outliers were identified using DFDIST and BAYSESCAN. DFDIST is a modification for dominant markers of the software developed by Beaumont and Nichols [38]. DFDIST estimates a distribution of observed *F*_ST_ versus *uH*_E_, and loci under selection were identified by comparing them to a simulated neutral distribution. DFDIST parameters include critical frequency = 0.99; Zhivotovsky parameter = 0.25; trimmed mean *F*_ST_ = 0.3 (excluding 30% of highest and 30% of lowest *F*_ST_ values); smoothing proportion = 0.04; 500,000 resamplings; and critical *p* = 0.05. AFLP Loci with observed *F*_ST_ against *uH*_E_ falling above the 95% confidence level of simulated distribution were recognized as *F*_ST_ outliers under directional selection. BAYESCAN v.2.1 [39] uses a reversible-jump Markov chain Monte Carlo algorithm to estimate the ratio of posterior probabilities of selection over neutrality (the posterior odds (POs)). Two hundred pilot runs of 50,000 iterations followed by a sample size of 50,000 with a thinning interval of 20 among 10^6^ iterations were performed in BAYESCAN. Selection is detected when locus-specific component (α) is significantly different from zero. A positive α suggests divergent selection, while negative values suggest balancing or purifying selection. We used a criterion of a logarithmic scale of log_10_ (PO) > 2 as decisive evidence, corresponding to posterior probabilities between 0.99 and 1 [40], for selection over neutrality for a locus under directional selection (α > 0).

### 2.6. Test for Associations of AFLP Loci with Environmental Variables

To assess the associations of all genetic loci with environmental variables, the latent factor mixed model (LFMM) [41] and Samβada [42] were employed in testing for significant correlations of genetic variation in each AFLP locus with environmental variables. A latent random factor was incorporated in the hierarchical Bayesian mixed effect model implemented in the LFMM. Considering the background level of the population structure due to the demographic history and isolation-by-distance pattern, a number of latent factors of 3 was used according to the DAPC result (see Results), and a matrix of genetic variation was used as a fixed factor. For each environmental predictor, ten LFMM runs with 10,000 iterations of the Gibbs sampling algorithm and a burn-in period of 5000 cycles were performed. We obtained Z-scores for each environmental predictor by combining the results of ten independent LFMM runs and *p* values adjusted using the genomic inflation factor (*λ*) [41]. A false discovery rate (FDR)-adjusted *p* value of 1% was further applied using the *qvalue* function of the R package qvalue [43]. Samβada was used to assess the correlations of allele frequencies of AFLP loci with values of environmental variables based on the multiple univariate logistic regression approach. A 1% FDR for *p* value adjustment for both Wald and G scores was used to assess the fit of the model with environmental variables against the null model without environmental variables.

A Bayesian logistic regression analysis implemented in the *stan_glm* function of the R package rstanarm [44] was employed to further justify the associations of the potential *F*_ST_ outliers, identified using both BAYESCAN and DFDIST, with environmental variables. In *stan_glm,* the weakly informative priors following Student’s *t* distribution with a mean of zero and seven degrees of freedom were used, and the scale of the prior distribution was 10 for the intercept and 2.5 for the predictors. All *stan_glm* models were run with four chains for 2000 warm-up and 2000 sampling steps. The *posterior_interval* function of the R package rstanarm was employed to estimate 95% credible intervals for the determination of significant correlations of potential *F*_ST_ outliers with environmental variables. In *stan_glm* analysis, the effective sample size values representing overall sampling efficiencies for each predictor estimated were between 1632.8 and 7199.4, and the convergence diagnostic statistics were all close to 1. These values indicate good priors applied and stable estimates obtained.

### 2.7. Relative Contribution of Environmental Variables Explaining Variation in Potential F_ST_ Outliers

To test for the most important environmental variables explaining outlier genetic variation, we used the *forward.sel* function of the R package adespatial [45]. Forward selection was stopped if either the conventional level of significance (*p* < 0.05) or the global adjusted *R*^2^ was exceeded to prevent the overestimation of the explained variance, and significance was determined based on 999 permutations. Environmental variables in the three environmental categories were analyzed separately, and variables explaining more than 5% of outlier genetic variation (adjusted *R*^2^ > 0.05) were retained as the final set of environmental variables in the study (Table S2). Variables most importantly influencing outlier genetic variation were assessed using functions within the R package MuMIn [46]. Generalized linear models (GLMs) with a logit link function and a binomial residual distribution were used to assess the relationships between the outlier variation and the final set of retained environmental variables, and McFadden’s pseudo-*R*^2^ for the fixed effect of the best predicting model explaining outlier variation was calculated using the *pR2* function of the R package pscl [47]. GLMs that fit all possible models for each outlier (response variable) were used in the *dredge* function and the subsequent model averaging analyses based on the Akaike information criterion with a correction for small sample sizes (AICc) (ΔAICc ≤ 2, the *model.avg* function). The AICc was used to rank the models and to calculate the Akaike sum of weights (SW) for each model [48]. The SW index, calculated using the *importance* function, was used to assess the relative importance of environmental variables contributing to explaining variation in the outlier loci. However, the SW index is arguably not an appropriate measure, representing the importance of model selection [49]. Therefore, a 95% confidence interval (CI) for each environmental variable included in the best predicting model for the variation in each outlier was estimated. The allele frequencies of *F*_ST_ outliers were used to test for correlation with the population mean *F*_ST_.

### 2.8. Mantel Test and Variation Partitioning

The mantel test was used to analyze the correlations of the outlier AFLP Euclidean distance matrix with the Euclidean distance matrix of environments using the *mantel* function of the R package vegan [50] and the Euclidean distance matrix of environments controlling for latitudinal difference using the *mantel.partial* function. Environmental variables and outlier variation were used in a redundancy analysis (RDA). An RDA estimates the relative contribution of environmental variables explaining the outlier variation using the *varpart* function of the R package vegan. The total outlier variation was partitioned into four fractions attributable to (a) a pure environmental effect, (b) a geographically structured environmental effect, (c) a pure geographic effect, and (d) a residual effect [51]. We tested the significance of these fractions using the *anova.cca* function of the R package vegan with 999 permutations. Sample site geographic coordinates were used as geographic effects in variation partitioning.

## 3. Results

### 3.1. Genetic Diversity and Structure

A total of 621 AFLP (mean ± SD: 60.09 ± 13.96) loci was obtained using 11 selective amplification primer combinations (Table S1). The percentage of polymorphism varied from 20.7 (population EFS) to 48.7 (population HDD), with a mean of 37.0 (Table 2). The average level of *uH*_E_ was 0.123, ranging from 0.102 in population TRK to 0.151 in population BTWS. A strong departure from random association between AFLP loci based on the measures of multilocus LD, *I*_A_, and *r*_D_ was found for all populations examined (Table 2). The linear mixed effect model (LMM) analysis showed significant differences in the mean *uH*_E_ per locus among populations (*χ*^2^ = 116.2, *p* < 0.001) and in many between-population comparisons (Table S4). Using the total data, population differentiation was high based on the AMOVA (*Φ*_ST_ = 0.308, *p* < 0.001) (Table S5) and pairwise *F*_ST_ (average *F*_ST_ = 0.298, *p* < 0.0001) (Table S6).

### 3.2. Genetic Clustering and Relationships

**Figure 2:**
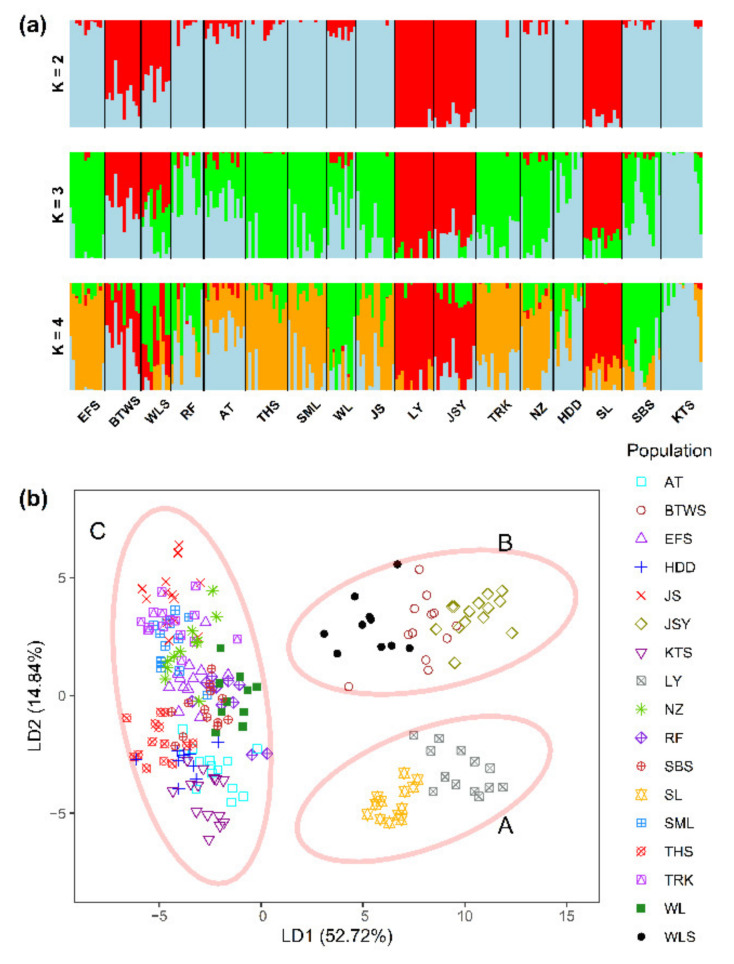
Analysis of genetic homogeneous groups of 212 individuals of *Zingiber kawagoii* based on the total AFLP variation using LEA (**a**) and DAPC (**b**). The clustering scenarios for *K* = 2–4 were displayed in LEA. The two linear discriminants LD1 and LD2 of DAPC described 52.72% and 14.84% of the total AFLP variation, respectively.

**Figure 3:**
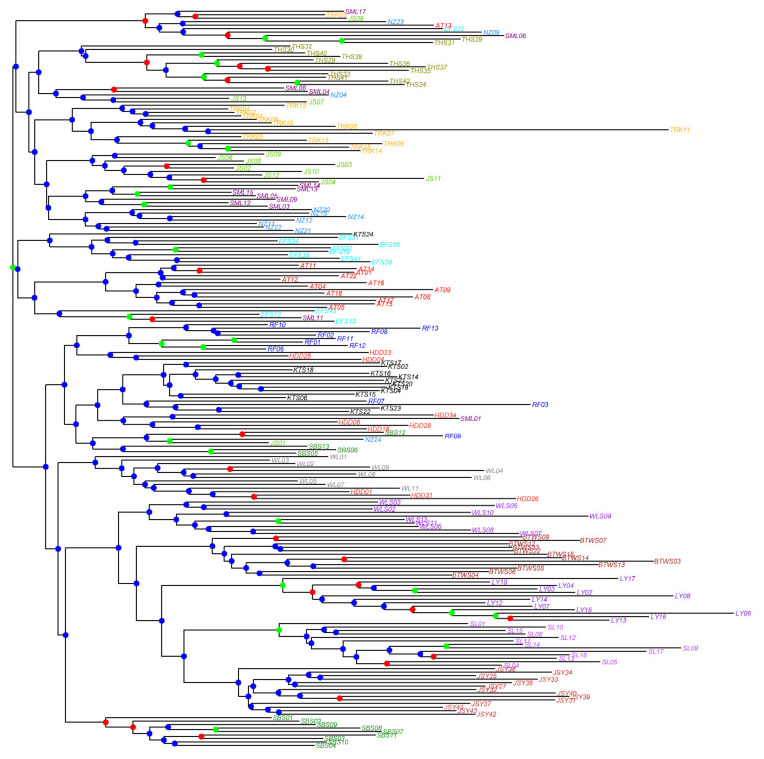
Neighbor-joining tree of 212 individuals of *Zingiber kawagoii* based on Nei’s genetic distances calculated using the total AFLP variation. Branch tip labels for individuals of different populations are colored differently. For each node, bootstrap support values greater than 70%, between 50% and 70%, and smaller than 50% are coded with green, red, and blue, respectively.

The mean minimal CE in LEA (Figure S1a) and the BIC in DAPC (Figure S1b) were minimized at *K* = 18 and *K* = 8, respectively. However, we observed changes in both the mean minimal CE and BIC elbowed at *K* = 3, which is consistent with three genetically homogeneous groups observed in LEA and DAPC (Figure 2). The three genetic clusters revealed by DAPC were: cluster A, containing populations LY and SL; cluster B, containing populations JSY, BTWS, and WLS; and cluster C, containing populations AT, EFS, HDD, JS, KTS, NZ, RF, SBS, SML, THS, TRK, and WL (Figure 2b). Genetic homogeneous grouping is concordant when comparing LEA to DAPC, despite the gene flow between populations observed in the LEA result (Figure 2a). Individuals of DAPC clusters A and B were grouped together in the NJ tree (Figure 3). Individuals of different populations of DAPC clusters A and B were clearly separated into different clades in the NJ tree, albeit with low BSPs. However, individuals of populations grouped in DAPC cluster C showed intermingled relationships in the NJ tree.

### 3.3. Latitudinal Trend of Annual Temperature Range and Population Mean F_ST_

**Figure 4:**
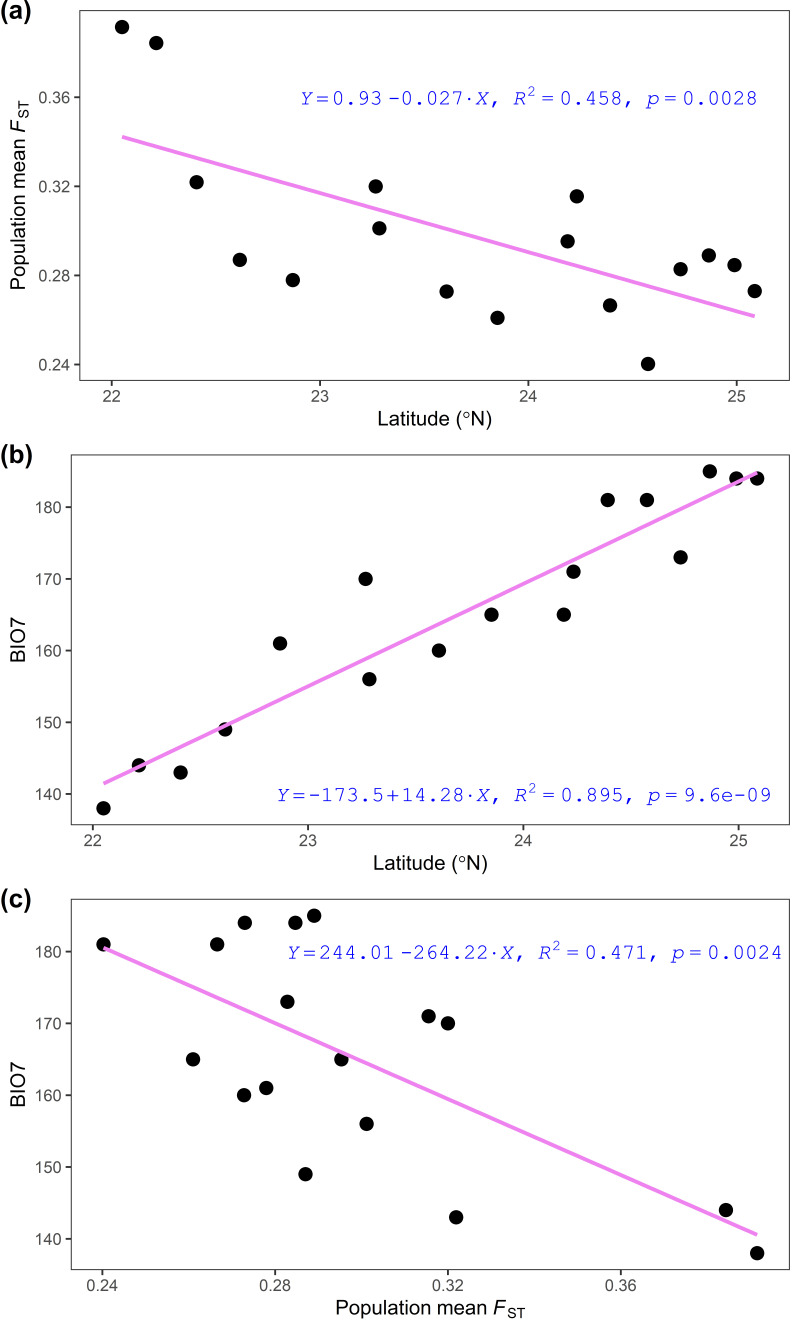
Regression plots showing the relationships between population mean *F*_ST_ and latitude (**a**), between annual temperature range and latitude (**b**), and between annual mean temperature and population mean *F*_ST_ (**c**). Pairwise population *F*_ST_ was estimated using the total AFLP variation and used in calculation of population mean *F*_ST_ (Table S6) BIO7, annual temperature range.

Pearson’s correlation test found a significant negative relationship between the population mean *F*_ST_ and latitude (Table S7, Figure 4a) and a significant positive relationship between the annual temperature range and latitude (Table S7, Figure 4b). Therefore, a moderate negative correlation between the annual temperature range and population mean *F*_ST_ was found (Figure 4c). Significant relationships between the population mean *F*_ST_, latitude, and annual temperature range were also observed when the LY population was excluded from the analysis (population mean *F*_ST_ vs. latitude: Pearson’s *r* = −0.5842, *p* = 0.01748; population mean *F*_ST_ vs. annual temperature range: Pearson’s *r* = −0.5786, *p* = 0.0189; latitude vs. annual temperature range: Pearson’s *r* = 0.9341, *p* < 0.0001).

### 3.4. F_ST_ Outliers and Relative Importance of Environmental Variables Explaining Outlier Variation

BAYESCAN and DFDIST identified 26 loci (4.18%) as potential *F*_ST_ outliers (Table 3). All 26 *F*_ST_ outliers identified by *F*_ST_-based methods were strongly correlated with environmental variables assessed using Samβada, the LFMM, and rstanarm (Table 3). We found very high population genetic differentiation based on the outlier data using the AMOVA (*Φ*_ST_ = 0.628, *p* < 0.001; Table S5). Additionally, the annual temperature range and annual precipitation were the two most important environmental variables influencing outlier genetic variation (adjusted *R*^2^ = 0.192 and adjusted *R*^2^ = 0.098, respectively; Table 4).

The relative importance of environmental variables estimated using model averaging of the most parsimonious models (ΔAICc ≤ 2) and the 95% CIs for coefficients of environmental covariates in the best predicting models revealed that environmental variables acted in a combinatorial fashion, influencing the genetic variations in the 26 *F*_ST_ outliers (Table 5). Although the annual temperature range was not necessarily included in the best predicting models explaining outlier variation (Table 5), it was the only environmental variable significantly correlated with latitude (Table S7, Figure 4b).

**Figure 5:**
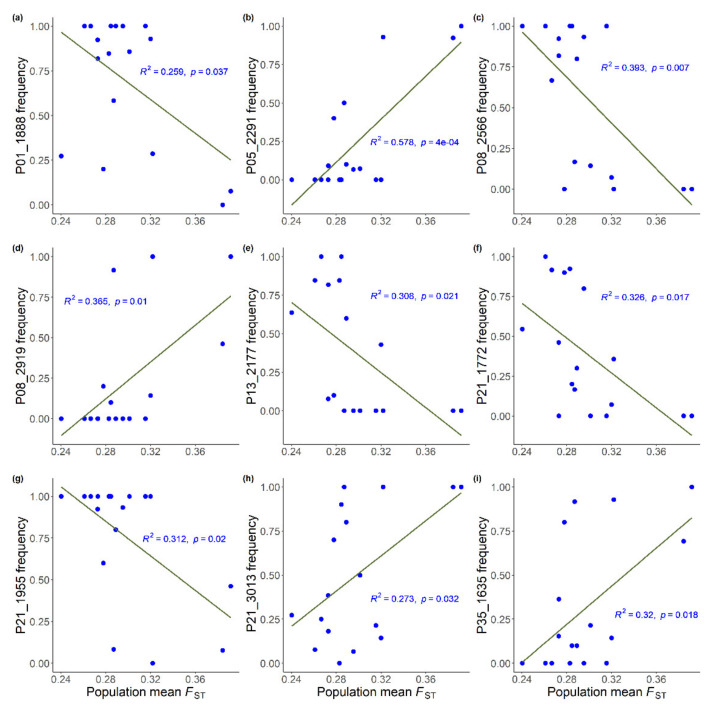
Linear regression plots of nine *F*_ST_ outliers showing significant correlation relationships of allele frequency with population mean *F*_ST_. Pearson’s correlation test results are reported in Table S8.

The 95% CIs indicated that no environmental variable significantly explained genetic variations in three outlier AFLP loci (P12_1612, P19_2619, and P19_2812) (Table 5), whereas the other 23 *F*_ST_ outliers were significantly explained by a combination of environmental variables. Moreover, environmental variables with high SW values may show no significant effects (95% CIs bracket zeros) on outlier variation. Nine outlier loci were found to have either significant positive (P05_2291, P08_2919, P21_3013, and P35_1635) or negative (P01_1888, P08_2566, P13_2177, P21_1772, and P21_1955) relationships of allele frequency with the population mean *F*_ST_ (Figure 5). However, we found no allele frequency correlation with latitude (Table S8).

The total explainable outlier genetic variation by the seven retained environmental variables was 47.4% based on the 26 outlier loci, of which 24.0% and 18.9% were, respectively, attributed to pure environmental and geographically structured environmental effects. However, only 4.5% was attributed purely to geographic effects (Table 6).

## 4. Discussion

### 4.1. Pattern of Adaptive Divergence along a Narrow Latitudinal Range

Biological species richness and speciation rate are higher toward the equator, which may have been related to the higher genetic divergence and greater evolutionary independence of populations within species [5]. Clinal variation may arise from neutral drift processes or adaptation linked to local environmentally associated genotypes [52]. This study found no correlation between genetic diversity (*uH*_E_) and latitude (Pearson’s *r* = 0.021, *p* = 0.936; Table S7). However, a strong negative correlation between the latitude and population mean *F*_ST_ (Pearson’s *r* = −0.677, *p* = 0.003) indicates higher levels of population genetic divergence in the low-latitude populations of *Z. kawagoii* (Figure 4a, Table S7). Additionally, a strong positive correlation of the annual temperature range with latitude (Pearson’s *r* = 0.946, *p* < 0.001; Figure 4b, Table S7) is consistent with an environmental gradient along latitude. Correlations between the latitude and population mean *F*_ST_ and between the latitude and annual temperature range are evidence for local adaptation.

The LY population, located on a small island situated off the southeast coast of Taiwan, had the lowest genetic distance to the population JSY in southern Taiwan (*F*_ST_ = 0.300; Table S6) but clustered with the geographically closest SL population in DPAC (Figure 1 and Figure 2b). Additionally, the LY population was closely related to other geographic proximity populations in southern Taiwan, including JSY, BTWS, and WLS based on the NJ tree (Figure 1 and Figure 3). These results suggest genetic connectivity between populations across the sea barrier, which was also found in *Pemphis acidula* [53] and *Setaria viridis* [54]. Additionally, the direction and significant relationships between the population mean *F*_ST_, latitude, and annual temperature range held when the LY population was excluded based on Pearson’s correlation test (see results). Thus, we included the LY population as one of the populations in the *Z. kawagoii* latitudinal distribution.

Multilocus LD (*I*_A_ and *r*_D_, Table 1) measures the non-random association of alleles [26,27]. A significant departure from zero of these measures may result from recent bottlenecks because of mating among genetically close individuals within populations [55], but it can also be influenced by mutation, recombination, natural selection, genetic drift, gene flow, and population size [56]. Our findings of environmentally dependent genetic variation (Table 3 and Table 5) and changes in the allele frequencies of adaptive loci strongly correlated with population divergence (Figure 5) suggest that significant *I*_A_ and *r*_D_ detected in all populations examined (Table 2) could be owing in part to natural selection [14,57,58]. In this study, the exceptionally high level of population differentiation analyzed using the 26 adaptive loci (*Φ*_ST_ = 0.628, *p* < 0.001; Table S5) suggests that environmentally based divergent selection may have played important roles in generating population adaptive divergence (the mantel test of outlier genetic distance matrix against environmental distance matrix: *r*_M_ = 0.505, *p* = 0.001). We identified environmental factors (the seven retained environmental variables, Table 1) significantly correlated with outlier genetic variation controlling for the latitudinal effect (partial mantel test: *r*_M_ = 0.464, *p* = 0.001), suggesting that multiple environmental factors impose as selective drivers for local adaptation (Table 3 and Table 5). The strong adaptive differentiation can be attributed to the complexity of environmental factors, causing micro-evolutionary differentiation between *Z. kawagoii* populations [12,14,52].

### 4.2. Latitudinal Cline of Annual Temperature Range Is the Major Selective Driver for Local Adaptation

Temperature and precipitation are the two most important selective drivers for local adaptation in plants commonly found to influence fitness-related traits and survival [2,59]. In this study, the annual mean temperature was excluded from the final set of environmental variables, and it was not significantly correlated with the population mean *F*_ST_ (Pearson’s *r* = 0.116, *p* = 0.659) or with the latitude (Pearson’s *r* = 0.133, *p* = 0.611). The annual temperature range with a higher adjusted *R*^2^ than other environmental variables (Table 4) may have played the main role in driving outlier genetic variation, resulting in a significant latitudinal pattern (Figure 4b). The differential combinatorial effects of environmental factors [60] in the best predicting models also played crucial roles in influencing genetic variations in the 26 *F*_ST_ outliers, with high pseudo *R*^2^ values (Table 5). The co-optimizing environmental variables may invoke locally adaptive genetic variation, particularly in rugged topographic landscapes such as Taiwan.

A strong linear fit of annual temperature range to a trend of reduction in the population mean *F*_ST_ along latitude (Figure 4) is consistent with Martin and McKay [5], who found that lower-latitude populations displayed greater genetic divergence. Local genotypes may be better adapted to local conditions, and adapted gene frequencies increase as natural selection persists overtime, which may generate clines in allele frequencies along the environmental gradient [10,61]. The nine potential *F*_ST_ outliers displayed strong correlations of allele frequencies with the population mean *F*_ST_ (Figure 5), suggesting the strong impact of selective pressures on population differentiation and the maintenance of adaptive integrity against the opposing forces of maladapted gene flow [10,62].

Adaptive divergence associated with thermal plasticity has been observed in natural populations of *Plantago* *lanceolata* [63] and *Cynodon dactylon* [64] along large-scale latitudinal gradients. However, a study finding annual thermal plasticity associated with population adaptive divergence along small-scale latitudinal gradients, to our knowledge, has not been documented. Thermal plasticity was thought to be more adaptive at higher latitudes due to the greater thermal variation in higher latitudes for species distributed in large geographic scales [65]. However, our results suggest adaptive evolution is evoked in response to different thermal ranges at higher- and lower-latitude populations (Figure 5).

### 4.3. Not Only Leading- but Also Trailing-Edge Populations Are Important for Zingiber kawagoii Conservation

In the current context of climate change, ecological, evolutionary, and conservation studies have demonstrated that populations at both limits of species distribution range evolved distinct genetic and phenotypic features [4,12]. Selection along thermal gradients of the environment can lead to the local adaptation and acclimatization of thermal-tolerance limits among populations [66,67]. The result of adaptive population differentiation might have related to range expansion toward higher latitudes (Figure 4 and Figure 5), and environmental boundaries between populations sharply shaped latitudinal cline in genetic divergence [10].

An initiation of shifting poleward in latitude and upward in elevation after the LGM and under the current global warming is expected [1]. The degree of temperature variability can affect the thermal-tolerance margins of organisms and is crucial to locally adapted responses to warming [66,67]. The current level of genetic diversity is a key determinant of a population adapting to changing environments [2,4,10,14]. High-diversity populations have broader stress-mitigation responses than low-diversity populations [68]. However, the genetic diversity of *Z. kawagoii* estimated using AFLP was lower (average *uH*_E_ = 0.123) than that of the Brazilian *Z. officinale* (average *uH*_E_ = 0.312) [69], three Indian *Zingiber* species (*Z. neesanum*: 0.240; *Z. nimmonii*: 0.164, and *Z. zerumbet*: 0.367) [70], and the diversity of thirteen plant species (average *uH*_E_ = 0.230) [71]. The relatively low level of genetic diversity in *Z. kawagoii* was also reflected in the low average percentage of polymorphism (Table 2) and low cpDNA variation [6]. The low population genetic diversity *in Z. kawagoii* (Table 2) is probably due to factors such as the nature of inbreeding [7] and the isolation of populations [2,3], which may reduce the potential of evolving local adaptation [57,59].

Although low-latitude rear edge populations are expected to be small in size and are hence characterized by low genetic diversity [4], our data do not meet this rear edge hypothesis (Pearson’s correlation test between *uH*_E_ and latitude: *r* = 0.021, *p* = 0.936). It is likely that geographic variation in thermal tolerance limits, consisting of both spatial temperature gradient and warming, can influence the rate of range shifts [4]. While cool margins are expanding, warm margin populations may persist locally due in part to local topographic and ecological conditions [1,4]. Moreover, two contrasting patterns of allele frequency change which correlated strongly with population divergence (Figure 5) indicate an increase in the probability of the presence of adaptive loci associated with the leading- and trailing-edge environments (Table 3 and Table 5). Apart from the nine loci (Figure 5), other outlier AFLP loci may persist at intermediate frequencies (Figure S2) for long periods due to heterogeneous selective pressures. Spatial range expansions can generate allele frequency gradients attributed to distinct selective processes [72]. This study suggests that locally adapted trailing- and leading-edge populations (Figure 5) are important for the future survival of species such as *Z. kawagoii*.

## 5. Conclusions

We studied the population mean *F*_ST_ along a latitudinal gradient that extended from the south of the *Z.* *kawagoii* distribution to its northern distributional margin. The annual temperature range related to the thermal tolerance of local populations could be the major environmental factor influencing outlier genetic variation. Additionally, the combination of various environmental variables may also be important to the local adaptation of *Z.* *kawagoii*. This study identified the presence of natural selection acting on adaptive loci at a small latitudinal scale, contributing to understanding how herbaceous species respond to environmental changes. The results broaden the generality of the latitudinal population divergence which is closely linked to environmental gradients in both the latitudinal leading- and trailing-edge populations of *Z. kawagoii*. Ecological speciation may occur in the low-latitude populations of *Z. kawagoii,* particularly because of high genetic divergence against other populations with narrower thermal tolerance limits.

## Figures and Tables

**Table 1:** The seven retained site environmental variables of the 17 populations of *Zingiber* *kawagoii*.

Population	Aspect	BIO7	BIO12	NDVI	PET	RH	WS_mean_
Antong (AT)	288.05	15.6	1933	0.84	1380.86	78.21	3.15
Beitawushan (BTWS)	120.11	14.9	4616	0.77	1497.83	76.68	2.69
Erfenshan (EFS)	314.41	18.1	2494	0.85	1634.22	78.23	2.73
Huangdidian (HDD)	299.16	18.4	3481	0.84	1419.22	78.88	2.57
Jianshi (JS)	164.95	17.3	2539	0.86	1436.64	78.91	2.51
Jinshuiying (JSY)	80.97	14.3	4749	0.80	1449.96	75.92	2.65
Kantoushan (KTS)	290.69	17.0	3120	0.78	1622.86	78.69	2.69
Lanyu (LY)	235.58	13.8	2760	0.77	1379.87	87.58	7.45
Nanzhuang (NZ)	269.41	18.1	2564	0.84	1489.51	78.67	2.60
Ruifang (RF)	250.43	18.4	3282	0.77	1398.84	78.29	2.83
Shibishan (SBS)	81.66	16.0	2726	0.77	1855.08	81.43	2.24
Shuangliu (SL)	63.68	14.4	3100	0.86	1830.68	76.15	2.96
Sunmoonlake (SML)	256.65	16.5	2262	0.80	1757.00	81.12	1.28
Tahsueshan (THS)	323.05	17.1	2569	0.81	1632.20	78.31	2.54
Taroko (TRK)	294.93	16.5	2292	0.84	1453.29	78.78	2.84
Wulai (WL)	105.67	18.5	3231	0.78	1477.30	78.82	2.43
Weiliaoshan (WLS)	358.40	16.1	3093	0.84	1769.88	77.34	2.78

Aspect (0–360°). BIO7, annual temperature range (°C); BIO12, annual precipitation (mm); NDVI, normalized difference vegetation index (unitless); PET, annual total potential evapotranspiration (kg/m^2^/year); RH, relative humidity (%); WSmean, mean wind speed (m/s).

**Table 2:** Site properties and genetic parameters of the 17 sampled populations of *Zingiber kawagoii* estimated based on the total AFLP variation.

Population	Latitude Longitude	Altitude (m)	*N*	%*P*	*uH*_E_ (SE)	*I*_A_ (*p*)	*r*_D_ (*p*)
Antong (AT)	23.2847 121.3721	610	14	35.2	0.113 (0.006)	3.614 (0.001)	0.016 (0.001)
Beitawushan (BTWS)	22.6148 120.7022	1192	12	40.7	0.151 (0.007)	2.040 (0.001)	0.008 (0.001)
Erfenshan (EFS)	24.3919 120.8240	769	12	20.7	0.115 (0.007)	1.753 (0.001)	0.009 (0.001)
Huangdidian (HDD)	24.9894 121.6799	432	10	48.7	0.143 (0.007)	2.022 (0.001)	0.009 (0.001)
Jianshi (JS)	24.7307 121.2895	850	13	32.2	0.105 (0.006)	3.914 (0.001)	0.023 (0.001)
Jinshuiying (JSY)	22.4075 120.7564	1488	14	36.6	0.126 (0.006)	1.137 (0.001)	0.005 (0.001)
Kantoushan (KTS)	23.2671 120.5010	583	14	35.4	0.115 (0.006)	4.421 (0.001)	0.022 (0.001)
Lanyu (LY)	22.0496 121.5257	302	13	33.9	0.123 (0.007)	6.455 (0.001)	0.031 (0.001)
Nanzhuang (NZ)	24.5742 121.0436	467	11	45.5	0.126 (0.006)	6.256 (0.001)	0.029 (0.001)
Ruifang (RF)	25.0861 121.8385	349	11	43.6	0.131 (0.007)	9.336 (0.001)	0.045 (0.001)
Shibishan (SBS)	23.6077 120.7045	1347	13	37.5	0.125 (0.006)	2.517 (0.001)	0.012 (0.001)
Shuangliu (SL)	22.2140 120.7961	255	13	30.0	0.103 (0.006)	2.489 (0.001)	0.014 (0.001)
Sunmoonlake (SML)	23.8519 120.8982	816	13	33.1	0.115 (0.006)	7.748 (0.001)	0.036 (0.001)
Tahsueshan (THS)	24.2326 120.9003	937	14	33.4	0.103 (0.006)	4.739 (0.001)	0.027 (0.001)
Taroko (TRK)	24.1880 121.6382	929	15	31.0	0.102 (0.006)	7.054 (0.001)	0.034 (0.001)
Wulai (WL)	24.8663 121.5498	143	10	46.7	0.145 (0.007)	4.942 (0.001)	0.022 (0.001)
Weiliaoshan (WLS)	22.8695 120.6571	694	10	44.3	0.144 (0.007)	2.460 (0.001)	0.011 (0.001)
Average			12.5	37.0	0.123 (0.006)		

*N*, number of samples used; *%P*, the percentage of polymorphic loci; *uH*_E,_ unbiased expected heterozygosity; *I*_A_, index of association; *r*_D_, modified index of association.

**Table 3:** *F*_ST_ outliers identified via BAYESCAN and DFDIST and strongly associated with environmental variables. Codes below the environmental columns (aspect, BIO7, BIO12, NDVI, PET, RH, and WS_mean_) represent strong correlations between *F*_ST_ outliers and environmental variables identified using LFMM (L), Samβada (S), and rstanarm (R).

Locus	DFDIST *F*_ST_	BAYESCAN log_10_ (PO)	Aspect	BIO7	BIO12	NDVI	PET	RH	WS_mean_
P01_1612	0.329	1000	LSR	LS	S		LSR	R	L
P01_1888	0.372	1000	SR	SR	S			R	R
P01_2213	0.397	1000	SR	LSR	S	R	R	SR	
P03_1760	0.340	1000		SR	L	LS	R		R
P03_1890	0.406	1000	SR	LSR		R			SR
P03_2200	0.345	1000			LSR			R	R
P03_3475	0.291	1000		LSR	S	R	L		LSR
P05_2291	0.346	1000	R	LR				R	R
P08_2566	0.493	1000		LSR	S		R	R	SR
P08_2919	0.400	1000	SR	LSR	SR	S	L	R	LR
P12_1612	0.259	2.164	R	R					
P12_1956	0.323	1000			LSR			R	
P12_2591	0.344	1000		R	LSR	S		L	
P13_1855	0.407	1000	R						
P13_2177	0.452	1000		LSR				R	SR
P13_2234	0.434	1000	SR	SR	LSR		R		R
P13_2991	0.339	1000			LSR	R	LS		
P19_2111	0.487	1000		R	SR	SR		R	SR
P19_2619	0.384	1000	LSR	LSR	SR			SR	
P19_2812	0.239	2.657	S		LSR	S		S	
P21_1772	0.384	1000	R	R	R	LSR		R	SR
P21_1865	0.413	1000		R	LSR	SR		LR	R
P21_1955	0.407	1000	SR	LSR	SR			R	R
P21_3013	0.366	1000	SR	SR	SR	R	L	R	LR
P35_1635	0.361	1000	S	LSR	SR	S	R	R	R
P35_2014	0.382	1000		R		R	R	R	R

Aspect (0–360°). BIO7, annual temperature range (°C); BIO12, annual precipitation (mm); NDVI, normalized difference vegetation index (unitless); PET, annual total potential evapotranspiration (kg/m^2^/year); RH, relative humidity (%); WSmean, mean wind speed (m/s).

**Table 4:** Relative contribution (adjusted *R*^2^) and *F* test of environmental variables explaining outlier genetic variation in *Zingiber kawagoii* using a forward selection procedure.

Environmental Variable	Adjusted *R*^2^	Cumulative Adjusted *R*^2^	*F* Value (*p*)
BIO7	0.1916	0.1916	51.00 (0.001)
BIO12	0.0984	0.2900	30.11 (0.001)
NDVI	0.0374	0.3724	13.68 (0.001)
RH	0.0315	0.3589	11.09 (0.001)
WS_mean_	0.0298	0.3887	10.16 (0.001)
PET	0.0287	0.4174	11.63 (0.001)
Aspect	0.0118	0.4292	5.27 (0.001)

Aspect (0–360°). BIO7, annual temperature range (°C); BIO12, annual precipitation (mm); NDVI, normalized difference vegetation index (unitless); PET, annual total potential evapotranspiration (kg/m^2^/year); RH, relative humidity (%); WSmean, mean wind speed (m/s).

**Table 5:** Relative importance and significance of environmental variables explaining variations in the 26 *F*_ST_ outliers based on model averaging using MuMIn. Numbers in parentheses are the Akaike sum of weights (SW) of each environmental variable across all parsimonious predicting models (ΔAICc ≤ 2). In bold, variables receiving strong support (i.e., the 95% confidence interval did not overlap with zero). McFadden’s pseudo *R*^2^ was calculated with the variables (predictors) selected as the best model with the lowest AICc used in the generalized linear model. For variables that are part of the best model with the lowest AICc, the sign of regression coefficient is shown: +, positive; −, negative.

Locus	Pseudo *R*^2^	Aspect	BIO7	BIO12	NDVI	PET	RH	WS_mean_
P01_1612	0.475	0.55 (8)	0.5 (7)	**0.93 (14)+**	0.29 (5)	**1 (15)+**	0.32 (5)	0.81 (11)+
P01_1888	0.387	**1 (4)+**	0.18 (2)	0.21 (2)	0.78 (3) −	**1 (4) −**	**1 (4)+**	**1 (4) −**
P01_2213	0.560	**1 (3)+**	**1 (3) −**	0.21 (1)		0.25 (1)	**1 (3) −**	**1 (3) −**
P03_1760	0.248	**1 (3) −**	0.18 (1)	**1 (3) −**		**1 (3) −**	**1 (3) −**	0.41 (1) −
P03_1890	0.364	**1 (5)+**	**1 (5)+**	0.51 (2) −	0.86 (4) −	0.15 (1)	0.13 (1)	**1 (5) −**
P03_2200	0.197	**1 (3) −**		**1 (3) −**	0.65 (2)	**1 (3) −**	0.66 (2)+	**1 (3) −**
P03_3475	0.726	1 (2)+	1 (2) −		**1 (2) −**		**0.38 (1)**	0.62 (1) −
P05_2291	0.643	1 (5)	0.11 (1)	1 (5)+	0.26 (1)	0.21 (1)	**1 (5) −**	**1 (5)+**
P08_2566	0.646	**0.83 (2)+**	**0.17 (1)**	**0.53 (2)**	0.47 (1)+	**1 (3) −**	**0.47 (1)+**	**1 (3) −**
P08_2919	0.749		**0.26 (10)**	**1 (3)+**	0.21 (1)	**0.74 (2)+**		1 (3)+
P12_1612	1.000	0.2 (1)	1 (5)+	1 (5) −	0.2 (1)	0.2 (1) −	0.2 (1)	0.2 (1)
P12_1956	0.189		**1 (3)+**	**1 (3)+**	0.66 (2) −		0.18 (1)	
P12_2591	0.362	**1 (3)+**	0.24 (1)	**1 (3)+**	**1 (3) −**	**1 (3) −**	0.25 (1)	**1 (3) −**
P13_1855	0.582	**1 (3) −**	**1 (3)+**	0.24 (1)	**1 (3)+**	**1 (3)+**	0.23 (1)	**1 (3)+**
P13_2177	0.494	**1 (2)+**	**1 (2)+**	**1 (2)+**	**1 (2)**	0.25 (1)	**1 (2)+**	**1 (2) −**
P13_2234	0.272	0.38 (2)	0.34 (2)	**1 (5) −**	**1 (5) −**	**1 (5) −**	0.12 (1)	**1 (5) −**
P13_2991	0.614		**1 (3)+**	**1 (3) −**	**1 (3) −**	**1 (3) −**	0.23 (1)	0.24 (1)
P19_2111	0.587	0.32 (2)	0.47 (2)	**1 (4) −**	**1 (4)+**	**1 (4) −**	**1 (4)+**	**1 (4) −**
P19_2619	0.829	0.08 (1)	0.07 (1)	0.26 (3)	0.24 (3)	0.57 (7) −	0.92 (10)+	0.17 (2)
P19_2812	0.846	0.2 (1)	0.2 (1)	0.8 (4)+	0.4 (2) −	0.2 (1)	0.2 (1)	
P21_1772	0.295	0.34 (2)	0.3 (2)		**1 (6)+**	0.11 (1)	0.13 (1)	**1 (6) −**
P21_1865	0.326	0.23 (1)	**1 (3)+**	**1 (3)+**	**1 (3) −**		0.21 (1)	1 (3) −
P21_1955	0.652	0.63 (5)+	**0.59 (5)**	**1 (8) −**	0.6 (5)	0.59 (5) −	0.49 (4)+	**0.5 (4) −**
P21_3013	0.374	0.78 (2) −	**1 (3) −**	**1 (3)+**	**1 (3)+**		0.21 (1)	1 (3)+
P35_1635	0.534	0.08 (1)	0.56 (5)	**1 (8)+**	0.18 (2)	**0.92 (7)+**	0.4 (3)	**1 (8)+**
P35_2014	0.211	0.85 (4) −	0.29 (2)	**1 (5) −**	**1 (5) −**	**1 (5) −**	**1 (5) −**	0.4 (2)

Aspect (0–360°). BIO7, annual temperature range (°C); BIO12, annual precipitation (mm); NDVI, normalized difference vegetation index (unitless); PET, annual total potential evapotranspiration (kg/m^2^/year); RH, relative humidity (%); WSmean, mean wind speed (m/s).

**Table 6:** The percentage of variation (adjusted *R*^2^)-explained outlier variation and variation accounted for by non-geographically structured environmental variables [a], shared (geographically structured) environmental variables [b], pure geographic factors [c], and undetermined component [d] analyzed based on variations in 26 *F*_ST_ outliers. Fraction [a+b+c] represents total explainable variation.

	Adjusted *R*^2^ (Percentage of Total Explainable Variation)	*F*	*p*
Environment [a]	0.240 (50.6%)	14.66	0.001
Environment + Geography [b]	0.189 (39.9%)		
Geography [c]	0.045 (9.5%)	9.76	0.001
[a+b+c]	0.474	22.16	0.001
Residual [d]	0.526		

Environmental variables used in [a] were aspect; Geographic variable for [c] was calculated using geographical coordinates of sample sites.

## Data Availability

The data presented in this study are available on request from the corresponding author.

## Supplementary Materials

The following are available online at: https://www.mdpi.com/article/10.3390/plants11192490/s1, Figure S1: Minimum cross-entropy (a) and Bayesian information criteria (b) for evaluation of clustering scenarios, respectively, analyzed using LEA and DAPC. Figure S2: Heatmap of allele frequencies of the 26 outlier loci identified. The sequence of populations was arranged according to degree of latitude (°N). Table S1: Primer combinations, number of markers, and error rate per locus in AFLP for investigation in Zingiber kawagoii. Table S2: Relative contribution (adjusted R2) and F test of environmental variables explaining outlier genetic variation in Zingiber kawagoii using a forward selection procedure. Table S3: Variance inflation factor (VIF) of the seven environmental variables and Pearson’s correlation coefficients between these variables. Table S4: Summary of Tukey’s post hoc pairwise population comparisons of the mean unbiased expected heterozygosity (uHE) per locus using a linear mixed effect model. In linear mixed effect model, population was treated as a fixed factor and locus as a random factor based on the total AFLP variation of Zingiber kawagoii populations. Table S5: Genetic differentiation between populations within species of the 17 populations of Zingiber kawagoii based on the total and outlier AFLP variation using analysis of molecular variance (AMOVA). Table S6: Pairwise FST between populations of Zingiber kawagoii based on the total AFLP data using ARLEQUIN with 10,000 permutations. All pairwise comparisons were found to be significant (*p* < 0.0001). See Table 1 for population code. Table S7: Summary of the results of Pearson’s correlation test of population mean FST and seven environmental variables against population latitude. Table S8: Summary of the results of Pearson’s correlation test of allele frequencies of the 26 outlier loci against population mean *F*_ST_ and against population latitude.
