# Supplementary material for: Pattern of Adaptive Divergence in Zingiber kawagoii Hayata (Zingiberaceae) along a Narrow Latitudinal Range
Source: Plants (Basel). 2022 Sep 23;11(19):2490. doi: 10.3390/plants11192490 (PMC9573048; doi:10.3390/plants11192490)
Supplement: Supplementary file 1 [file plants-11-02490-s001.zip › plants-1877115-supplementary.pdf]

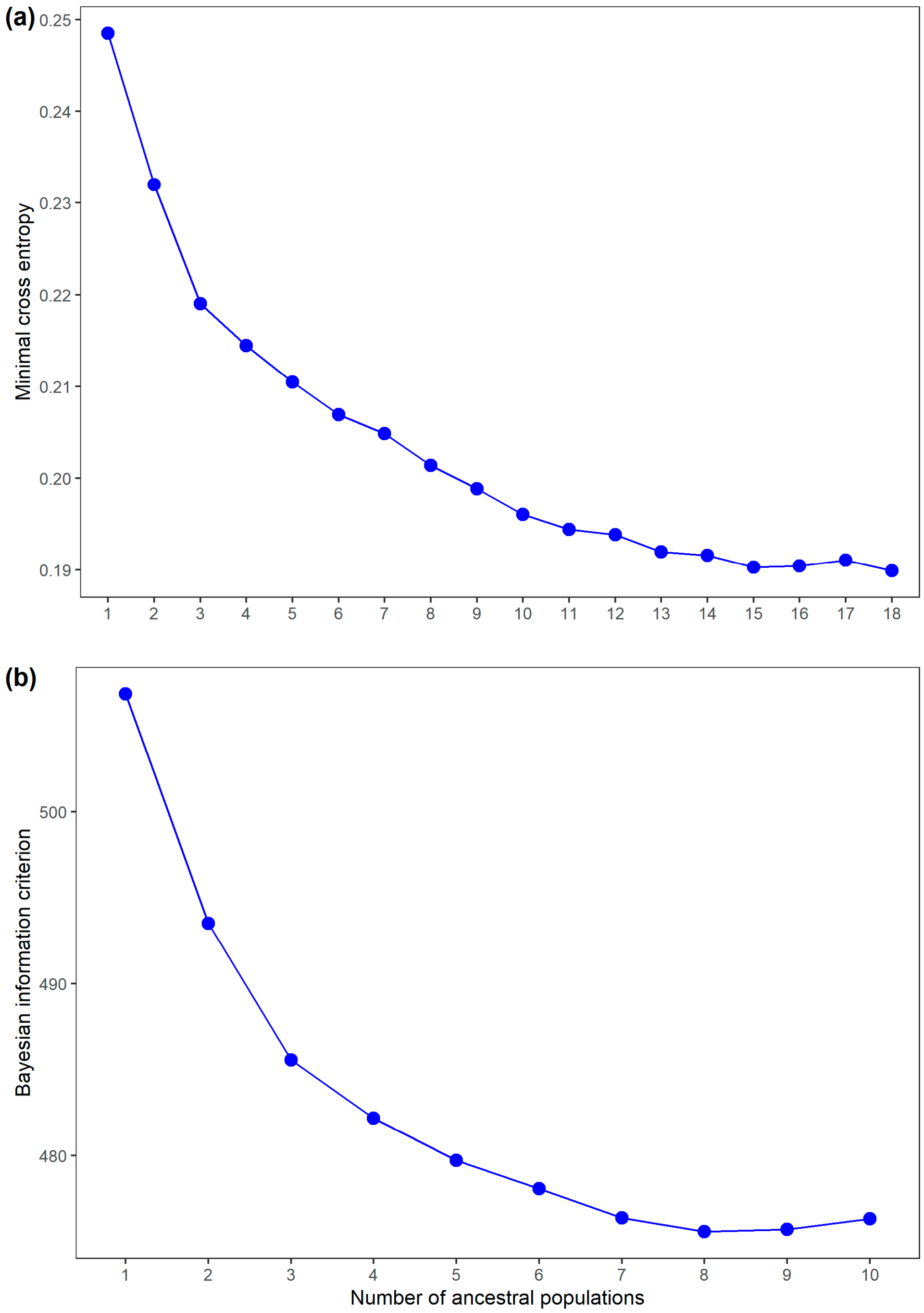

Figure S1. Minimum cross-entropy (**a**) and Bayesian information criterion (**b**) for evaluation of clustering scenarios, respectively, analyzed using LEA and DAPC.

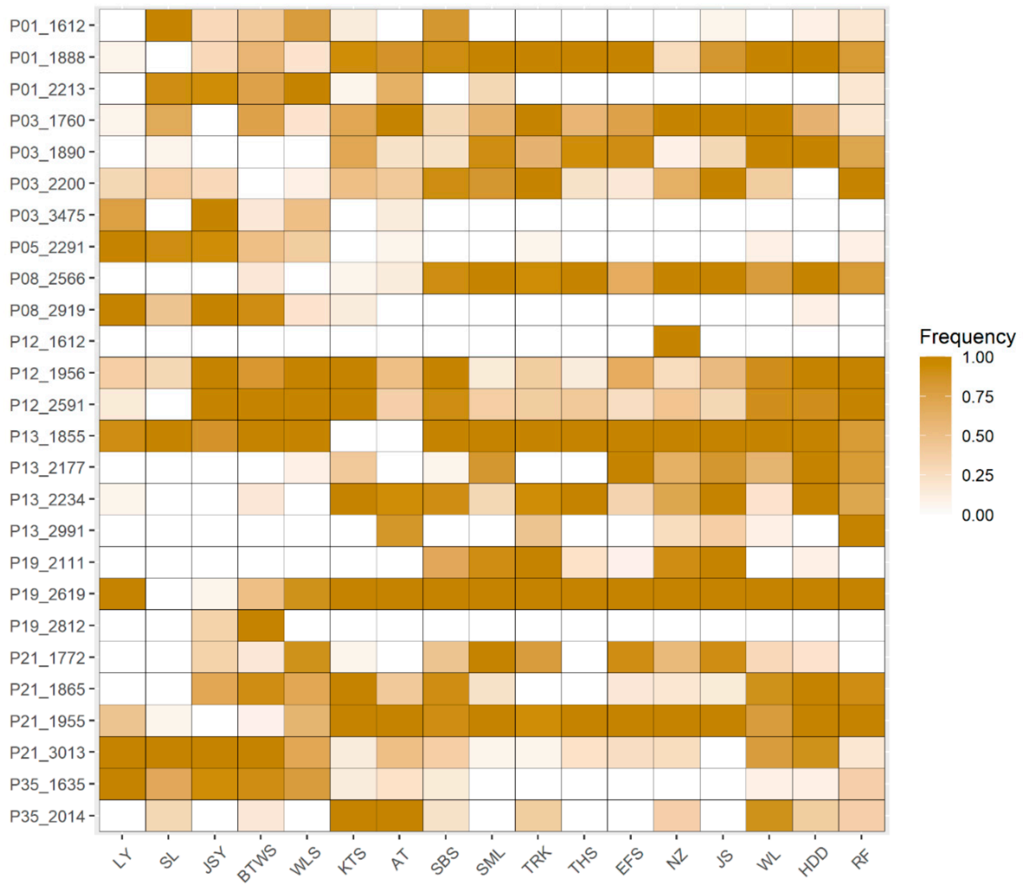

Figure S2. Heatmap of allele frequencies of the 26 outlier loci identified. The sequence of populations was arranged according to degree of latitude (°N).

Table S1. Primer combinations, number of markers, and error rate per locus in AFLP for investigation in *Zingiber kawagoii*.

Primer combination	Numbers of markers	Error rate (%)
1 *Eco*RI + AAC - *Mse*I + CTCGG	72	4.83
2 *Eco*RI + AAC - *Mse*I + CTCGT	62	3.31
3 *Eco*RI + AAC - *Mse*I + CTCCA	59	3.63
4 *Eco*RI + AAC - *Mse*I + CTCTC	74	2.95
5 *Eco*RI + AAC - *Mse*I + CTGAG	64	3.98
6 *Eco*RI + AAC - *Mse*I + GAC	76	3.47
7 *Eco*RI + AAC - *Mse*I + GAT	69	4.80
8 *Eco*RI + AAC - *Mse*I + GTC	58	4.44
9 *Eco*RI + AAC - *Mse*I + TCC	55	4.49
10 *Eco*RI + TAA - *Mse*I + GAT	42	4.41
11 *Eco*RI + TAA - *Mse*I + GCA	30	4.87
Average	60.09	4.11

Table S2. Relative contribution (adjusted R^2) and F test of environmental variables explaining outlier genetic variation of *Zingiber kawagoii* using a forward selection procedure.

Category of environmental variables	Adjusted R^2	Cumulative adjusted R^2	F value (P)
Bioclimate			
BIO7	0.1916	0.1916	51.00 (0.001)
BIO12	0.0984	0.2900	30.11 (0.001)
BIO9	0.0395	0.3294	13.30 (0.001)
Topography			
Elevation	0.1394	0.1394	28.54 (0.001)
Aspect	0.0518	0.1912	11.83 (0.001)
Slope	0.0431	0.2343	10.45 (0.001)
Ecology			
WSmean	0.0742	0.742	17.91 (0.001)
RH	0.0916	0.1658	24.06 (0.001)
NDVI	0.0668	0.2326	19.20 (0.001)
PET	0.0539	0.2865	16.71 (0.001)
Soil pH	0.0290	0.3154	9.76 (0.001)
LAI	0.0294	0.3448	10.24 (0.001)
MI	0.0302	0.3750	10.89 (0.001)
EVI	0.0217	0.3967	8.34 (0.001)

Aspect (0–360°) and slope (0–90°). BIO7, annual temperature range; BIO9, mean temperature of the driest quarter; BIO12, annual precipitation; EVI, enhanced vegetation index; LAI, leaf area index; MI, annual moisture index; NDVI, normalized difference vegetation index; PET, annual total potential evapotranspiration; RH, relative humidity; WS_{mean}*, mean wind speed.*

Table S3. Variance inflation factor (VIF) of the seven environmental variables and Pearson's correlation coefficients between these variables.

Variable	VIF	Aspect	NDVI	PET	RH	WS_{mean}	BIO7
Aspect	1.766						
NDVI	2.224	0.250					
PET	1.599	-0.159	0.040				
RH	4.602	0.137	-0.379	-0.111			
WS_{mean}	4.352	0.057	-0.239	-0.395	0.681		
BIO7	2.235	0.432	0.195	-0.097	-0.164	-0.476	
BIO12	3.165	-0.519	-0.399	-0.119	-0.382	-0.029	-0.298

Aspect (0–360°); BIO7, annual temperature range; BIO12, annual precipitation; NDVI, normalized difference vegetation index, PET, annual total potential evapotranspiration; RH, relative humidity; WS_{mean}*, mean wind speed.*

Table S4. Summary of Tukey's post-hoc pairwise population comparisons of the mean unbiased expected heterozygosity (uH_E) per locus using a linear mixed effect model. In linear mixed effect model, population was treated as a fixed factor and locus as a random factor based on the total AFLP variation of the *Zingiber kawagoii* populations.

Population pair	Estimate	SE	df	t	P
AT-BTWS	-0.036522	0.00731	10560	-4.996	0.0001
AT-EFS	0.001528	0.00731	10560	0.209	1
AT-HDD	-0.019867	0.00731	10560	-2.717	0.3438
AT-JS	0.013361	0.00731	10560	1.828	0.923
AT-JSY	-0.010507	0.00731	10560	-1.437	0.9919
AT-KTS	0.004871	0.00731	10560	0.666	1
AT-LY	-0.00138	0.00731	10560	-0.189	1
AT-NZ	-0.012531	0.00731	10560	-1.714	0.9551
AT-RF	-0.006865	0.00731	10560	-0.939	1
AT-SBS	-0.003979	0.00731	10560	-0.544	1
AT-SL	0.013943	0.00731	10560	1.907	0.8928
AT-SML	0.000482	0.00731	10560	0.066	1
AT-THS	0.011754	0.00731	10560	1.608	0.975
AT-TRK	0.011457	0.00731	10560	1.567	0.9805
AT-WL	-0.014623	0.00731	10560	-2	0.8492
AT-WLS	-0.021246	0.00731	10560	-2.906	0.2305
BTWS-EFS	0.038051	0.00731	10560	5.205	<.0001
BTWS-HDD	0.016655	0.00731	10560	2.278	0.671
BTWS-JS	0.049883	0.00731	10560	6.823	<.0001

BTWS-JSY	0.026016	0.00731	10560	3.558	0.0361
BTWS-KTS	0.041394	0.00731	10560	5.662	<.0001
BTWS-LY	0.035143	0.00731	10560	4.807	0.0002
BTWS-NZ	0.023991	0.00731	10560	3.281	0.0863
BTWS-RF	0.029657	0.00731	10560	4.057	0.0057
BTWS-SBS	0.032544	0.00731	10560	4.451	0.0011
BTWS-SL	0.050465	0.00731	10560	6.903	<.0001
BTWS-SML	0.037005	0.00731	10560	5.062	0.0001
BTWS-THS	0.048276	0.00731	10560	6.603	<.0001
BTWS-TRK	0.047979	0.00731	10560	6.563	<.0001
BTWS-WL	0.021899	0.00731	10560	2.995	0.1865
BTWS-WLS	0.015277	0.00731	10560	2.09	0.7989
EFS-HDD	-0.021396	0.00731	10560	-2.927	0.2199
EFS-JS	0.011833	0.00731	10560	1.619	0.9733
EFS-JSY	-0.012035	0.00731	10560	-1.646	0.9688
EFS-KTS	0.003343	0.00731	10560	0.457	1
EFS-LY	-0.002908	0.00731	10560	-0.398	1
EFS-NZ	-0.01406	0.00731	10560	-1.923	0.886
EFS-RF	-0.008394	0.00731	10560	-1.148	0.9994
EFS-SBS	-0.005507	0.00731	10560	-0.753	1
EFS-SL	0.012415	0.00731	10560	1.698	0.9586
EFS-SML	-0.001046	0.00731	10560	-0.143	1
EFS-THS	0.010226	0.00731	10560	1.399	0.994
EFS-TRK	0.009928	0.00731	10560	1.358	0.9956
EFS-WL	-0.016151	0.00731	10560	-2.209	0.7206
EFS-WLS	-0.022774	0.00731	10560	-3.115	0.1374
HDD-JS	0.033228	0.00731	10560	4.545	0.0007
HDD-JSY	0.00936	0.00731	10560	1.28	0.9978
HDD-KTS	0.024739	0.00731	10560	3.384	0.0634
HDD-LY	0.018488	0.00731	10560	2.529	0.4796
HDD-NZ	0.007336	0.00731	10560	1.003	0.9999
HDD-RF	0.013002	0.00731	10560	1.778	0.9384
HDD-SBS	0.015889	0.00731	10560	2.173	0.7453
HDD-SL	0.03381	0.00731	10560	4.625	0.0005
HDD-SML	0.02035	0.00731	10560	2.783	0.3012
HDD-THS	0.031621	0.00731	10560	4.325	0.0019
HDD-TRK	0.031324	0.00731	10560	4.285	0.0022
HDD-WL	0.005244	0.00731	10560	0.717	1
HDD-WLS	-0.001379	0.00731	10560	-0.189	1
JS-JSY	-0.023868	0.00731	10560	-3.265	0.0906
JS-KTS	-0.00849	0.00731	10560	-1.161	0.9993

JS-LY	-0.014741	0.00731	10560	-2.016	0.8407
JS-NZ	-0.025893	0.00731	10560	-3.542	0.0382
JS-RF	-0.020226	0.00731	10560	-2.767	0.3118
JS-SBS	-0.01734	0.00731	10560	-2.372	0.6004
JS-SL	0.000582	0.00731	10560	0.08	1
JS-SML	-0.012879	0.00731	10560	-1.762	0.9431
JS-THS	-0.001607	0.00731	10560	-0.22	1
JS-TRK	-0.001904	0.00731	10560	-0.26	1
JS-WL	-0.027984	0.00731	10560	-3.828	0.0139
JS-WLS	-0.034607	0.00731	10560	-4.734	0.0003
JSY-KTS	0.015378	0.00731	10560	2.103	0.7904
JSY-LY	0.009127	0.00731	10560	1.248	0.9983
JSY-NZ	-0.002025	0.00731	10560	-0.277	1
JSY-RF	0.003642	0.00731	10560	0.498	1
JSY-SBS	0.006528	0.00731	10560	0.893	1
JSY-SL	0.02445	0.00731	10560	3.344	0.0716
JSY-SML	0.010989	0.00731	10560	1.503	0.9871
JSY-THS	0.022261	0.00731	10560	3.045	0.1648
JSY-TRK	0.021964	0.00731	10560	3.004	0.1825
JSY-WL	-0.004116	0.00731	10560	-0.563	1
JSY-WLS	-0.010739	0.00731	10560	-1.469	0.9898
KTS-LY	-0.006251	0.00731	10560	-0.855	1
KTS-NZ	-0.017403	0.00731	10560	-2.38	0.5937
KTS-RF	-0.011737	0.00731	10560	-1.605	0.9753
KTS-SBS	-0.00885	0.00731	10560	-1.211	0.9988
KTS-SL	0.009072	0.00731	10560	1.241	0.9985
KTS-SML	-0.004389	0.00731	10560	-0.6	1
KTS-THS	0.006883	0.00731	10560	0.941	1
KTS-TRK	0.006585	0.00731	10560	0.901	1
KTS-WL	-0.019494	0.00731	10560	-2.666	0.3787
KTS-WLS	-0.026117	0.00731	10560	-3.572	0.0345
LY-NZ	-0.011152	0.00731	10560	-1.525	0.985
LY-RF	-0.005486	0.00731	10560	-0.75	1
LY-SBS	-0.002599	0.00731	10560	-0.355	1
LY-SL	0.015323	0.00731	10560	2.096	0.7951
LY-SML	0.001862	0.00731	10560	0.255	1
LY-THS	0.013134	0.00731	10560	1.796	0.933
LY-TRK	0.012836	0.00731	10560	1.756	0.9447
LY-WL	-0.013243	0.00731	10560	-1.811	0.9283
LY-WLS	-0.019866	0.00731	10560	-2.717	0.3439
NZ-RF	0.005666	0.00731	10560	0.775	1

NZ-SBS	0.008553	0.00731	10560	1.17	0.9992
NZ-SL	0.026475	0.00731	10560	3.621	0.0292
NZ-SML	0.013014	0.00731	10560	1.78	0.9379
NZ-THS	0.024285	0.00731	10560	3.322	0.0766
NZ-TRK	0.023988	0.00731	10560	3.281	0.0864
NZ-WL	-0.002091	0.00731	10560	-0.286	1
NZ-WLS	-0.008714	0.00731	10560	-1.192	0.999
RF-SBS	0.002887	0.00731	10560	0.395	1
RF-SL	0.020808	0.00731	10560	2.846	0.2636
RF-SML	0.007348	0.00731	10560	1.005	0.9999
RF-THS	0.018619	0.00731	10560	2.547	0.466
RF-TRK	0.018322	0.00731	10560	2.506	0.4969
RF-WL	-0.007758	0.00731	10560	-1.061	0.9998
RF-WLS	-0.014381	0.00731	10560	-1.967	0.8658
SBS-SL	0.017922	0.00731	10560	2.451	0.539
SBS-SML	0.004461	0.00731	10560	0.61	1
SBS-THS	0.015733	0.00731	10560	2.152	0.7595
SBS-TRK	0.015435	0.00731	10560	2.111	0.7855
SBS-WL	-0.010644	0.00731	10560	-1.456	0.9907
SBS-WLS	-0.017267	0.00731	10560	-2.362	0.608
SL-SML	-0.013461	0.00731	10560	-1.841	0.9183
SL-THS	-0.002189	0.00731	10560	-0.299	1
SL-TRK	-0.002486	0.00731	10560	-0.34	1
SL-WL	-0.028566	0.00731	10560	-3.907	0.0103
SL-WLS	-0.035189	0.00731	10560	-4.813	0.0002
SML-THS	0.011272	0.00731	10560	1.542	0.9834
SML-TRK	0.010974	0.00731	10560	1.501	0.9873
SML-WL	-0.015105	0.00731	10560	-2.066	0.8128
SML-WLS	-0.021728	0.00731	10560	-2.972	0.1974
THS-TRK	-0.000297	0.00731	10560	-0.041	1
THS-WL	-0.026377	0.00731	10560	-3.608	0.0306
THS-WLS	-0.033	0.00731	10560	-4.514	0.0008
TRK-WL	-0.02608	0.00731	10560	-3.567	0.0351
TRK-WLS	-0.032703	0.00731	10560	-4.473	0.001
WL-WLS	-0.006623	0.00731	10560	-0.906	1

Table S5. Genetic differentiation between populations within species of the 17 populations of *Zingiber kawagoii* based on the total and outlier AFLP variation using analysis of molecular variance (AMOVA).

**Source of variation**	**df**	**Sum of square**	**Percent variation**	***Φ* Statistics (*P*)**
Total data				
Between populations	16	3547.087	30.75	Φ_{ST} = 0.3075 (0.0001)
Within populations	195	6617.465	69.25	
Total	211	10164.552	100	
Outlier data				
Between species	16	696.449	62.83	Φ_{ST} = 0.6283 (0.0001)
Within populations	195	384.830	37.17	
Total	211	1081.278	100	

Table S6. Pairwise F_{ST} between populations of *Zingiber kawagoii* based on the total AFLP data using ARLEQUIN with 10,000 permutations. All pairwise comparisons were found to be significant ($p < 0.0001$). See **Table 1** for population code.

	**AT**	**BTWS**	**EFS**	**HDD**	**JS**	**JSY**	**KTS**	**LY**	**NZ**	**RF**	**SBS**	**SL**	**SML**	**THS**	**TRK**	**WL**	**WLS**
	0.000																
BTWS	0.294	0.000															
EFS	0.262	0.330	0.000														
HDD	0.275	0.257	0.277	0.000													
JS	0.312	0.335	0.194	0.284	0.000												
JSY	0.338	0.150	0.350	0.354	0.371	0.000											
KTS	0.242	0.279	0.328	0.206	0.352	0.361	0.000										
LY	0.410	0.325	0.386	0.429	0.435	0.300	0.469	0.000									
NZ	0.227	0.275	0.157	0.246	0.151	0.316	0.296	0.371	0.000								
RF	0.276	0.248	0.276	0.154	0.240	0.329	0.224	0.407	0.247	0.000							
SBS	0.319	0.273	0.271	0.221	0.249	0.324	0.257	0.416	0.213	0.187	0.000						
SL	0.392	0.324	0.367	0.437	0.424	0.302	0.443	0.329	0.355	0.417	0.401	0.000					
SML	0.260	0.310	0.139	0.272	0.145	0.348	0.313	0.406	0.146	0.250	0.241	0.393	0.000				
THS	0.315	0.377	0.195	0.338	0.266	0.398	0.396	0.421	0.220	0.335	0.281	0.396	0.196	0.000			
TRK	0.262	0.352	0.208	0.301	0.176	0.392	0.330	0.438	0.154	0.280	0.292	0.419	0.199	0.268	0.000		
WL	0.317	0.259	0.267	0.203	0.289	0.299	0.293	0.407	0.231	0.246	0.201	0.426	0.285	0.325	0.334	0.000	
WLS	0.319	0.205	0.259	0.302	0.303	0.218	0.333	0.314	0.242	0.250	0.219	0.325	0.274	0.320	0.319	0.243	0.000
Average	0.301	0.287	0.265	0.285	0.283	0.322	0.320	0.392	0.240	0.273	0.273	0.384	0.261	0.316	0.295	0.289	0.278

Table S7. Summary of the results of Pearson's correlation test of population mean F_{ST} and seven environmental variables against population latitude.

Variable	Estimate	*P*
uH_E	0.021	0.936
Population mean F_{ST}	-0.677	0.003
Aspect	0.308	0.230
BIO7	0.946	< 0.001
BIO12	-0.326	0.202
NDVI	0.206	0.428
PET	-0.253	0.327
RH	-0.098	0.709
WSmean	-0.454	0.067

Table S8. Summary of the results of Pearson's correlation test of allele frequencies of the 26 outlier loci against population mean F_{ST} and against population latitude.

Locus	Population mean F_{ST}		Latitude	
	Estimate	*P*	Estimate	*P*
P01_1612	1.861	0.393	-0.090	0.289
P01_1888	-4.723	0.037	-0.087	0.356
P01_2213	2.409	0.343	0.002	0.985
P03_1760	-2.618	0.238	-0.003	0.973
P03_1890	-3.170	0.224	0.016	0.882
P03_2200	-2.331	0.310	-0.143	0.102
P03_3475	3.311	0.083	0.065	0.403
P05_2291	7.008	0.000	0.067	0.479
P08_2566	-7.015	0.007	-0.039	0.735
P08_2919	5.686	0.010	0.056	0.561
P12_1612	-2.257	0.142	0.061	0.322
P12_1956	-1.534	0.478	-0.140	0.084
P12_2591	-3.282	0.147	-0.112	0.212
P13_1855	-1.228	0.566	-0.040	0.635
P13_2177	-5.698	0.021	0.000	0.998
P13_2234	-3.509	0.189	-0.016	0.879
P13_2991	-1.794	0.386	-0.102	0.201
P19_2111	-4.768	0.066	-0.048	0.653
P19_2619	-3.960	0.052	-0.021	0.805
P19_2812	-0.092	0.956	-0.055	0.388
P21_1772	-5.471	0.017	-0.105	0.279
P21_1865	-3.020	0.250	-0.110	0.286
P21_1955	-5.175	0.020	-0.009	0.926
P21_3013	5.000	0.032	0.102	0.291
P35_1635	5.415	0.018	0.003	0.974
P35_2014	-0.006	0.998	0.004	0.964
